# Single-site laparoscopic ligation of the hernia sac in infants with congenital Morgagni hernia

**DOI:** 10.3389/fped.2023.1078244

**Published:** 2023-02-21

**Authors:** Cao Wang, Xiang Liu, Zhen Shu, Jia Yin, Zheng Luo, Guangxu Zhou, Bin Liu

**Affiliations:** ^1^ Department of Pediatric Surgery, Affiliated Hospital of Zunyi Medical University, Zunyi, China; ^2^ Department of Pediatric Surgery, Guizhou Children's Hospital, Zunyi, China

**Keywords:** congenital morgagni hernia, hernia sac high ligation, infants, single site laparoscopic, CMH

## Abstract

**Background:**

Congenital Morgagni hernia (CMH) is a rare midline defect involving herniation of abdominal viscera into the thoracic cavity through triangular parasternal gaps in the diaphragm.

**Methods:**

The medical records of three patients with CMH admitted to the Department of Pediatric Surgery at the Affiliated Hospital of Zunyi Medical University between 2018 and 2022 were retrospectively reviewed. Pre-operative diagnosis was based on chest x-ray, chest computerized tomography, and barium enema. All patients were treated with single-site laparoscopic ligation of the hernia sac.

**Results:**

Hernia repair was successful in all patients (males; age: 14 months, 30 months, 48 months). The average operative time for repair of a unilateral hernia was 20 ± 5 min. Volume of surgical blood loss was 2–3 ml. There was no damage to organs such as the liver or intestines, or to tissues such as the pericardium or the phrenic nerve. Patients were allowed a fluid diet 6–8 h after surgery, and remained on bed rest until 16 h after surgery. No postoperative complications occurred, and patients were discharged on postoperative Day 2 or 3. No symptoms or complications were noted during the 1–48 months of follow-up. Aesthetic outcomes were satisfactory.

**Conclusions:**

Single-site laparoscopic ligation of the hernia sac provides pediatric surgeons a safe and effective technique for repair of CMH in infants and children. The procedure is straightforward, operative time and surgical blood loss are minimal, recurrence is unlikely, and aesthetic outcomes are satisfactory.

## Background

Congenital Morgagni hernia (CMH), also known as Morgagni-Larrey hernia, is a rare midline defect involving herniation of abdominal viscera into the thoracic cavity through triangular parasternal gaps in the diaphragm. CMH, first described by Morgagni in 1761 (1), comprises 3%–5% of all types of congenital diaphragmatic herniae (2) and occurs in 1 in 4,800 live births (3). The etiology of CMH is multifactorial (4), and may include environmental exposures, genetic factors, malnutrition, Down syndrome and congenital heart disease (5) (6). CMH has no typical clinical symptoms and may be misdiagnosed. In some cases, CMH may present with respiratory and gastrointestinal symptoms, including acute respiratory distress in infancy (7) and acute or chronic intestinal obstruction, or intestinal perforation in adults. Surgical repair is the only treatment for CMH. Traditional approaches comprise laparotomy and open thoracic surgery (8). More recently, minimally invasive repairs of CMH in infants and children have used endoscopic and laparoscopic techniques (9), including the use of “U”-shaped stitches that transverse the abdominal wall, hernia sac, and the posterior rim of the diaphragmatic defect, and are secured in the subcutaneous tissue (10). Here, we describe a novel approach to the treatment of CMH, and describe our clinical experience in three cases that had satisfactory outcomes.

## Methods

**Figure 1:**
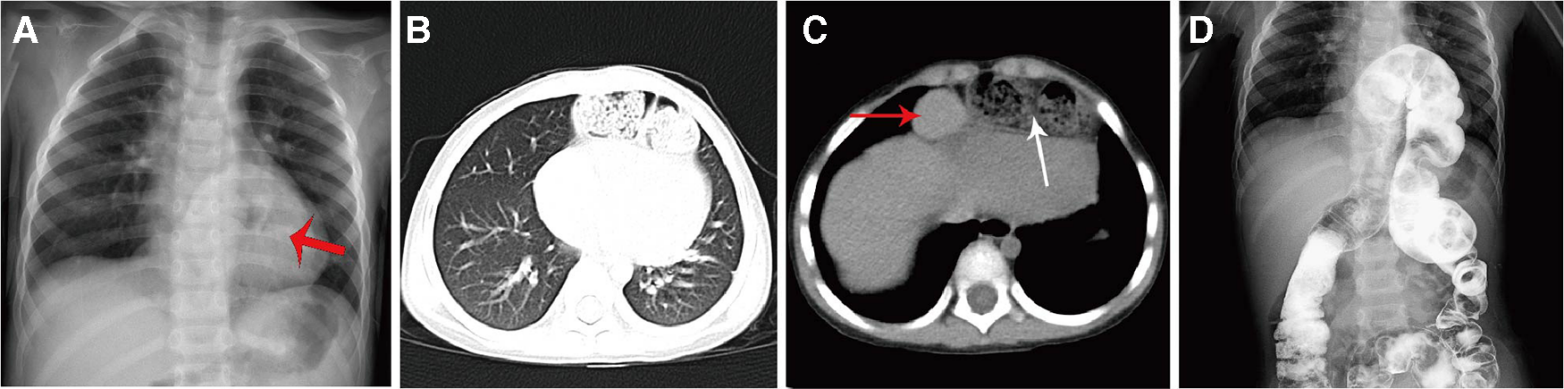
Preoperative imaging of a patient with a bilateral CMH. (**A**) Routine chest x-ray showing a left sided pericardial mediastinal shadow (red arrow); (**B**) Chest CT showing a shadow behind the sternum; (**C**) Chest CT showing partial herniation of the left lobe of the liver into the thoracic cavity, and herniation of the intestine into the thoracic cavity; (**D**) Barium enema showing herniation of the transverse colon above the diaphragm into the thoracic cavity.

The medical records of three patients with CMH admitted to the Department of Pediatric Surgery at the Affiliated Hospital of Zunyi Medical University between 2018 and 2022 were retrospectively reviewed. The patients were three males aged 14 months, 30 months, and 48 months. One patient presented with clinical symptoms of recurrent upper respiratory tract infection. One case was discovered incidentally when the patient was being treated for car accident injury. One case was discovered incidentally during investigations for chest deformity. Preoperatively, routine blood tests were normal in all patients. Routine chest x-rays were normal in two patients and showed increased density in the left lung field and a left sided pericardial mediastinal shadow in one patient (Figure 1A). In this patient, chest computerized tomography (CT) showed a bowel loop projecting into the anterior mediastinum, part of the left lobe of the liver and the colon projecting into the thoracic cavity, and cardiac compression (Figures 1B,C), and barium enema showed that a loop of the transverse colon was behind the sternum (Figure 1D). Angiography for upper gastrointestinal bleeding was negative and cardiac color ultrasound showed no abnormalities in all patients. The preoperative diagnosis was CMH. One patient had mild pectus carinatum that did not require surgical correction. The other two patients had no comorbidities. All patients were treated with single-site laparoscopic ligation of the hernia sac. Informed consent for the surgery was obtained from the parents of each infant.This study was approved by the ethics committee of our hospital.

**Figure 2:**
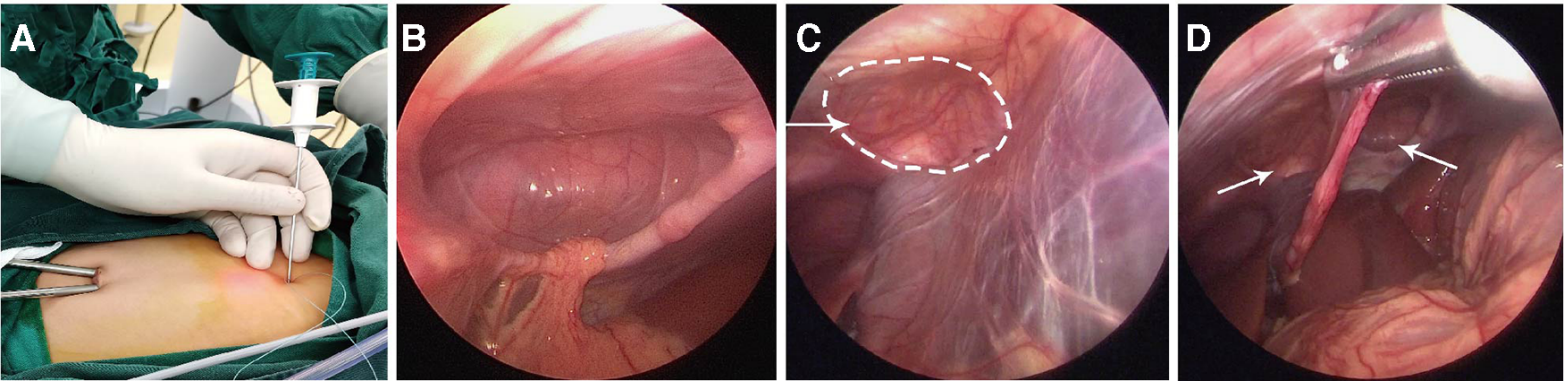
Single-site laparoscopic ligation of the hernia sac. (**A**) A left trocar was placed in the incision as an observation hole, followed by insertion of a 30°lenses. A right trocar was used as the operating hole. (**B**) Left side of the bilateral CMH, with the greater omentum forming the contents of the hernia sac. (**C**) The right side of the bilateral CMH was smaller than the left side (**D**) The bilateral CMH was behind the falciform ligament of the liver.

General endotracheal anesthesia was administered with the patient in the supine position. A 0.5 cm incision was made at the left and right edge of the umbilicus. A 0.5 cm trocar was placed through the incision, and the abdominal pressure was adjusted to 7–8 mmHg. A left trocar was placed in the incision as an observation hole, followed by insertion of a 30°lenses. A right trocar was used as the operating hole (Figure 2A). A U-shaped defect was found in all patients. This was confirmed as CMH, consistent with the preoperative diagnosis. One patient had a left-sided CMH, with the transverse colon and greater omentum forming the contents of the hernia sac. There was a 4 cm × 6 cm diaphragmatic defect. One patient had a bilateral CMH, with the greater omentum forming the contents of the hernia sac on the left side but not on the right side (Figures 2B–D). There was a 4 cm × 5 cm left diaphragmatic defect and a 3 × 4 cm right diaphragmatic defect. One patient had a right-sided CMH, and the hernia sac had no obvious contents. There was a 3 cm × 4 cm diaphragmatic defect.

**Figure 3:**
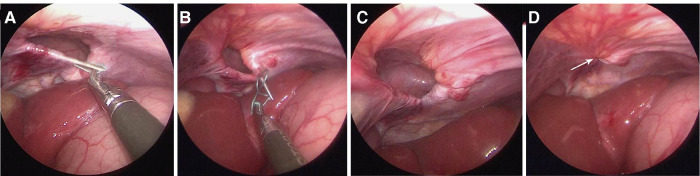
Operative procedure. (**A**) A needle holding threads of non-absorbable sutures (2–0 ethibond) was inserted at the midpoint of the diaphragmatic defect, introduced into the peritoneal space, and passed through the diaphragm into the abdominal cavity. One suture curved from the midpoint around the rim of the hernia sac. The needle was pulled out of the abdominal cavity leaving the suture inside; (**B**) The needle was reintroduced and a suture was curved in the opposite direction until it met the first suture at the midpoint of the posterior wall of the hernia sac; (**C**) The sutures were pulled out of the patient; (**D**) The sutures were tightened to ligate the hernia sac, and extracorporeal knots were tied (arrows).

The hernia sac contents were reduced. A needle holding threads of non-absorbable sutures (2–0 Ethibond) was inserted at the midpoint of the diaphragmatic defect, introduced into the peritoneal space, and passed through the diaphragm into the abdominal cavity. The suture curved from the midpoint around the rim of the hernia sac. The needle was pulled out of the abdominal cavity leaving the suture inside (Figure 3A). The needle was reintroduced and a second suture was curved in the opposite direction until it met the first suture at the midpoint of the posterior wall of the hernia sac (Figure 3B). The sutures were pulled out of the patient (Figure 3C), tightened to ligate the hernia sac, and extracorporeal knots were tied (Figure 3D). In the patient with a bilateral CMH, ligation was used to repair both sides of the hernia sac.

## Results

**Figure 4:**
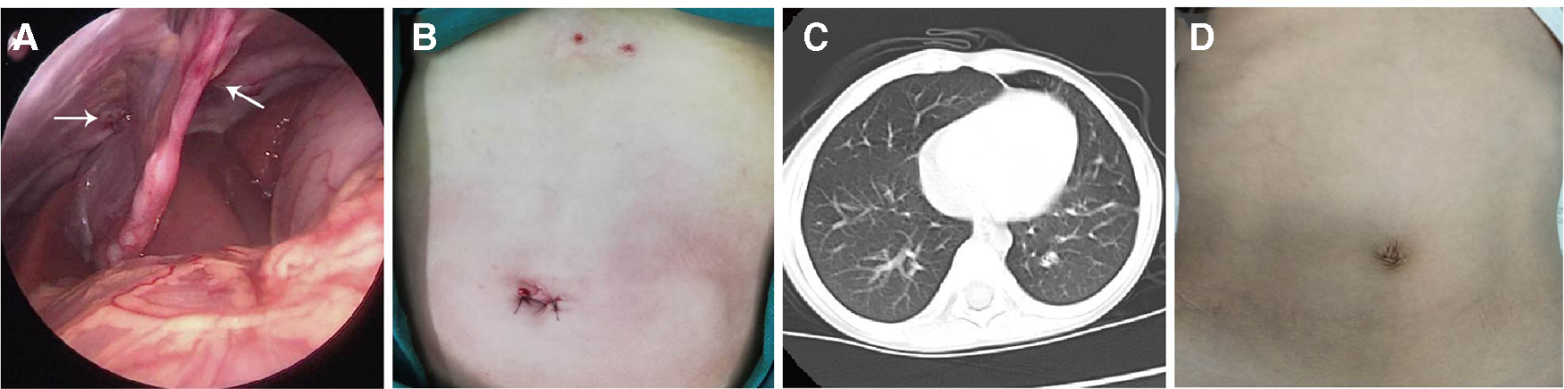
Postoperative outcomes. (**A**) Ligation of the bilateral hernia sac: ligation was firm and did not leave any gaps; (**B**) Abdominal incisions: the umbilical incision was small, and the extracorporeal knots were located under the xiphoid process; (**C**) Chest CT 15 months after the operation: the shadow behind the sternum had disappeared, and there was no aggravation of the pectus carinatum; (**D**) Aesthetic outcomes were satisfactory 15 months after the operation.

Hernia repair was successful in all patients. Ligation was a reliable technique, with no need for Optilene mesh. The average operative time for repair of a unilateral hernia was 20 ± 5 min. Volume of surgical blood loss was 2–3 ml. There was no damage to organs such as the liver or intestines, or to tissues such as the pericardium or the phrenic nerve. Resection of the falciform ligament of the liver was not required. The intestine was not pulled or compressed during surgery; therefore, patients were allowed a fluid diet 6–8 h after surgery, and remained on bed rest until 16 h after surgery. No postoperative complications occurred, and the patients were discharged on postoperative Day 2 or 3 (Table 1). No symptoms or complications were noted during the 1–48 months of follow-up. Chest CT showed no recurrence (Figure 4). There was no aggravation of the pectus carinatum in the affected patient. Aesthetic outcomes were satisfactory.

**Table 1:** Surgical outcomes for three patients with CMH.

Characteristic	Patient #1	Patient #2	Patient #3
Sex	Male	Male	Male
Age	14 months	30 months	48 months
Type of defect	Right	Left	Bilateral
Operative time	15 m	18 m	25 m
Volume of surgical blood loss	1 ml	2 ml	3 ml
Time to ingestion of solid food	7.5 h	6 h	8 h
Time on postoperative bed rest	16 h	16 h	16 h
Time to discharge	3 day	2 day	3 day

## Discussion

CMH occurs due to a failure of fusion or muscularization of the pars sternalis and pars costalis during development of the diaphragm, resulting in a triangular parasternal gap. An estimated 90% of CMH occurs on the right side; however, CMH may be left sided or bilateral (11, 12). In the present study, one patient had a right-sided CMH, one patient had a left-sided CMH, and one patient had a bilateral CMH.

CMH often lacks typical clinical manifestations and is prone to misdiagnosis and delayed diagnosis (7). Respiratory and gastrointestinal symptoms may be caused by an increase in intra-abdominal pressure and herniation of the abdominal contents into the chest cavity; therefore, symptoms may be precipitated by chronic constipation, chronic cough, trauma, pregnancy, and obesity (13). Hernia sac contents can include the colon, omentum, stomach, small intestine, or liver. Infants with CMH often present with respiratory distress syndrome (12). Late diagnosis in adults may occur in patients presenting with chest pain, acute or chronic intestinal obstruction, or intestinal perforation (6, 14, 15).

Most CHM are found incidentally, but CMH may be suspected in patients with recurrent respiratory infections, gastrointestinal symptoms or Down syndrome, with a diagnosis made radiologically. In the present study, one patient presented with clinical symptoms of recurrent upper respiratory tract infection. One case was discovered incidentally when the patient was being treated for car accident injury. One case was discovered incidentally during investigations for mild pectus carinatum; however, existing evidence suggests an association between pectus carinatum and CMH is unlikely. CMH may be seen on chest x-ray as an anterior mediastinal shadow on the lateral view. Chest CT may be required to confirm the diagnosis, as this can show a diaphragm defect and a retrosternal mass of fat density or an air-containing viscus when the omentum or bowel, respectively, have herniated into the chest cavity. Differential diagnosis includes a hiatus hernia, which can be excluded by upper gastrointestinal barium examination. Magnetic resonance imaging (MRI) may be employed, but the cost is usually prohibitive.

CMH should be treated surgically due to the risk of acute strangulation, volvulus, or incarceration. Open laparotomy or minimally invasive laparoscopy may be used (16). There is controversy over removal of the hernia sac. Resection of the hernia sac is technically challenging and can increase operative time and the risk of phrenic nerve injury, pneumothorax, pericardial injury, hemorrhage, and postoperative mediastinal emphysema (17). Mesh may be used in larger hernias and adults (18), but is not usually applicable in infants as the diaphragmatic defects are small and abdominal pressure is low.

Two main surgical methods are often used for repair of CMH: laparoscopic-assisted intralobular suture repair of the hernia sac (16) or percutaneous transperitoneal needle suture under laparoscopic surveillance (10, 14, 19, 20). Laparoscopic-assisted intralobular suture repair of the hernia sac is technically demanding and requires the insertion of 3 trocars in the abdominal wall, which may affect aesthetics. If the diaphragm at the anterior wall of the hernia sac lacks a firm edge, the diaphragm must be stitched to the abdominal wall, which is associated with a risk of recurrence. In percutaneous transperitoneal needle suture under laparoscopic surveillance (10, 14, 19, 20), such as U-stitches and mattress sutures, sutures of non-absorbable thread are placed through the abdominal wall into the peritoneal cavity, under the peritoneum and through the posterior edge of the hernia. The sutures are tied in the subcutaneous tissue to form a safe, effective and solid repair of the diaphragmatic defect. However, this approach requires several sutures placed through the full thickness of the abdominal wall, the technique is relatively complicated, and operative time is long. More recently, CMH has been successfully repaired using single-incision laparoscopic surgery and a suture-assisting needle (20), but this approach may not be cost-effective. In the present study, we used single-site laparoscopic ligation of the hernia sac, which was inspired by laparoscopic repair of infant and children with indirect inginal hernia. The technique does not require special instruments, the abdominal wall is punctured once, aesthetic outcomes are satisfactory, and the ligation is secure. The procedure is straightforward and can be performed by surgeons with experience treating indirect inguinal hernia in infants. If there is no adhesion between the hernia sac and the viscera, the operation may be completed by one experienced surgeon with one hole. If there is adhesion between the hernia sac and the omentum or intestine, an electric hook can be used to separate the adhesions. Operative time and surgical blood loss are minimal and no recurrence was detected with the longest follow-up time of 48 months.

## Conclusions

Single-site laparoscopic ligation of the hernia sac provides pediatric surgeons with a safe and effective technique for repair of CMH in infants and children. The procedure is straightforward, operative time and surgical blood loss are minimal, recurrence is unlikely, and aesthetic outcomes are satisfactory.

## Data Availability

The raw data supporting the conclusions of this article will be made available by the authors, without undue reservation.

## References

[1] Zani A Cozzi DA. Giovanni battista morgagni and his contribution to pediatric surgery. J Pediatr Surg. (2008) 43:729–33. 10.1016/j.jpedsurg.2007.12.065 18405723

[2] Van De Winkel N De Vogelaere K De Backer A Delvaux G. Laparoscopic repair of diaphragmatic morgagni hernia in children: review of 3 cases. J Pediatr Surg. (2011) 46(2):e23–6. 10.1016/j.jpedsurg.2010.10.005 21292066

[3] Ipek T Altinli E Yuceyar S Erturk S Eyuboglu E Akcal T. Laparoscopic repair of a morgagni-larrey hernia: report of three cases. Surg Today. (2002) 32:902–5. 10.1007/s005950200177 12376790

[4] Chandrasekharan PK Rawat M Madappa R Rothstein DH Lakshminrusimha S. Congenital diaphragmatic hernia—a review. Matern Health Neonatol Perinatol. (2017) 3:6. 10.1186/s40748-017-0045-1 28331629 PMC5356475

[5] Jetley NK Al-Assiri AH Al-Helal AS Al-Bin Ali AM. Down's syndrome as a factor in the diagnosis, management, and outcome in patients of morgagni hernia. J Pediatr Surg. (2011) 46:636–39. 10.1016/j.jpedsurg.2010.10.001 21496530

[6] Saqib SU Hamid K Chawla TU. Congenital morgagni hernia presenting as complete small bowel obstruction in the adult—a case report. Int J Surg Case Rep. (2020) 76:390–93. 10.1016/j.ijscr.2020.10.021 33086166 PMC7575642

[7] Alqahtani A Al-Salem AH. Laparoscopic-assisted versus open repair of morgagni hernia in infants and children. Surg Laparosc Endosc Percutan Tech. (2011) 21(1):46–9. 10.1097/SLE.0b013e318209021f 21304389

[8] Kiliç D Nadir A Döner E Kavukçu S Akal M Ozdemir N Transthoracic approach in surgical management of morgagni hernia. Eur J Cardiothorac Surg. (2001) 20:1016–9. 10.1016/s1010-7940(01)00934-4 11675191

[9] Danielson PD Chandler NM. Single-port laparoscopic repair of a morgagni diaphragmatic hernia in a pediatric patient: advancement in single-port technology allows effective intracorporeal suturing. J Pediatr Surg. (2010) 45(3):E21–4. 10.1016/j.jpedsurg.2009.12.029 20223304

[10] Akkoyun I Kececioglu M. A new, easy, and safe suturing technique for laparoscopic repair of morgagni hernias. J Pediatr Surg. (2012) 47:1626–8. 10.1016/j.jpedsurg.2012.04.007 22901931

[11] Al-Salem AH Zamakhshary M Al Mohaidly M Al-Qahtani A Abdulla MR Naga MI. Congenital Morgagni's hernia: a national multicenter study. J Pediatr Surg. (2014) 49:503–7. 10.1016/j.jpedsurg.2013.08.029 24726101

[12] Escarcega P Riquelme MA Lopez S Gonzalez AD Leon VY Garcia LR Multi-institution case series of pediatric patients with laparoscopic repair of morgagni hernia. J Laparoendosc Adv Surg Tech A. (2018) 28:1019–22. 10.1089/lap.2017.0621 29620946

[13] Karamustafaoglu YA Kuzucuoglu M Tarladacalisir T Yoruk Y. Transabdominal subcostal approach in surgical management of morgagni hernia. Eur J Cardiothorac Surg. (2011) 39:1009–11. 10.1016/j.ejcts.2010.09.031 21276731

[14] Furukawa H Wakasugi M Yoshikawa Y Sueda T Matsumura T Koga C Single-incision laparoscopic repair for a morgagni hernia: a case report. Asian J Endosc Surg. (2021) 14:124–27. 10.1111/ases.12823 33458961

[15] Mohamed M Al-Hillan A Shah J Zurkovsky E Asif A Hossain M. Symptomatic congenital morgagni hernia presenting as a chest pain: a case report. J Med Case Rep. (2020) 14:13. 10.1186/s13256-019-2336-9 31952551 PMC6969475

[16] Kuster GG Kline LE Garzo G. Diaphragmatic hernia through the foramen of morgagni: laparoscopic repair case report. J Laparoendosc Surg. (1992) 2(2):93–100. 10.1089/lps.1992.2.93 1534498

[17] Anadolulu AI Gercel G Kocaman OH. Laparoscopic repair of morgagni hernia in children. Ann Med Surg. (2020) 56:7–10. 10.1016/j.amsu.2020.05.012 PMC7292882 32551107

[18] Kuikel S Shrestha S Thapa S Maharjan N Kandel BP Lakhey PJ. Morgagni hernia in adult: a case report. Int J Surg Case Rep. (2021) 85:106286. 10.1016/j.ijscr.2021.106286 34388911 PMC8358627

[19] Zouari M Jallouli M Bendhaou M Zitouni H Mhiri R. Percutaneous suturing technique and single-site umbilical laparoscopic repair of a morgagni hernia: review of three cases. Arch Pediatr. (2015) 22:1272–5. 10.1016/j.arcped.2015.09.017 26552622

[20] Goto Y Mimori K Ogata S Shimizu H Fukuda Y Tanaka H. Single-incision laparoscopic full-thickness anterior abdominal wall repair of a morgagni hernia using a suture-assisting needle in a child: a case report. Asian J Endosc Surg. (2021) 14:548–52. 10.1111/ases.12864 32996277

